# Broadened immunity against influenza by vaccination with computationally designed influenza virus N1 neuraminidase constructs

**DOI:** 10.1038/s41541-018-0093-1

**Published:** 2018-11-29

**Authors:** E. R. Job, T. Ysenbaert, A. Smet, I. Christopoulou, T. Strugnell, E. O. Oloo, R. P. Oomen, H. Kleanthous, T. U. Vogel, X. Saelens

**Affiliations:** 1 VIB-UGent Center for Medical Biotechnology, VIB, 9052 Ghent, Belgium; 2 Department of Biomedical Molecular Biology, Ghent University, 9052 Ghent, Belgium; 3 Sanofi Pasteur, Research North America, Cambridge, Massachusetts USA

## Abstract

Split inactivated influenza vaccines remain one of the primary preventative strategies against severe influenza disease in the population. However, current vaccines are only effective against a limited number of matched strains. The need for broadly protective vaccines is acute due to the high mutational rate of influenza viruses and multiple strain variants in circulation at any one time. The neuraminidase (NA) glycoprotein expressed on the influenza virion surface has recently regained recognition as a valuable vaccine candidate. We sought to broaden the protection provided by NA within the N1 subtype by computationally engineering consensus NA sequences. Three NA antigens (NA5200, NA7900, NA9100) were designed based on sequence clusters encompassing three major groupings of NA sequence space; (i) H1N1 2009 pandemic and Swine H1N1, (ii) historical seasonal H1N1 and (iii) H1N1 viruses ranging from 1933 till current times. Recombinant NA proteins were produced as a vaccine and used in a mouse challenge model. The design of the protein dictated the protection provided against the challenge strains. NA5200 protected against H1N1 pdm09, a Swine isolate from 1998 and NIBRG-14 (H5N1). NA7900 protected against all seasonal H1N1 viruses tested, and NA9100 showed the broadest range of protection covering all N1 viruses tested. By passive transfer studies and serological assays, the protection provided by the cluster-based consensus (CBC) designs correlated to antibodies capable of mediating NA inhibition. Importantly, sera raised to the consensus NAs displayed a broader pattern of reactivity and protection than naturally occurring NAs, potentially supporting a predictive approach to antigen design.

## Influenza vaccines: Computational engineering of a broadly protective influenza vaccine

The high variability of the influenza virus — arising from its high mutation rate and wide range of strains — limits the effectiveness of influenza vaccines unless they induce a broad immune response, a difficult task when relying on natural viral antigens. Here, Xavier Saelens, Thorsten Vogel, Ray Oomen and colleagues applied a ‘cluster-based’ consensus computational approach to design three consensus sequences of the viral protein neuroaminidase (NA) subtype 1 that induce broadly protective immune responses in vaccinated mice. NA9100, a consensus NA sequence based on H1N1 virus strains collected from 1933 to today, was protective against all N1 viruses tested. By using a computational method to integrate multiple sequences of viral proteins into one consensus protein, the researchers provide a strategy that can be applied to develop broadly protective vaccine formulations for influenza virus.

## Introduction

Vaccination is the cornerstone of protection against influenza. Commercially available influenza vaccines are intended to antigenically match the hemagglutinin (HA) and neuraminidase (NA) of the prevailing circulating human influenza A and B strains. However, the virus constantly evolves under the immune pressure induced by seasonal influenza vaccines or natural infection, and this antigenic drift is most pronounced in the two surface glycoproteins HA and NA,^1^ the major immunogens of licensed influenza vaccines. This represents a burden for the influenza vaccine manufacturers because new vaccine strains (for H1N1, H3N2 and the two influenza B lineages) may have to be substituted into the vaccine for any given year. Furthermore, the predicted antigenic match between the vaccine strains and the actual circulating viruses is occasionally suboptimal, and this vaccine ‘mismatch’ is associated with reduced vaccine effectiveness.^2^ Thus, there is a need for broadly protective vaccination strategies which cannot only control current circulating strains, but which are also cross-reactive with antigenic variants that arise overtime.

Large efforts by multiple research groups have been put into designing broadly-reactive vaccine strategies. Some of these approaches are based on the use of viral antigens conserved across most or all influenza A subtypes. For example, the ectodomain of matrix protein 2 is largely conserved amongst avian and human influenza viruses, and as a vaccine antigen it can protect broadly against influenza viruses in experimental animal challenge models.^3^ Other groups have focused on raising antibodies to conserved regions of the HA stalk, which may provide protection against challenge with representative group I or group II influenza A viruses.^4,5^ Still other approaches aim at a subtype-specific (e.g., against all H1N1 or H5N1 viruses) broadening of protection based on computationally optimized consensus HA designs to elicit antibodies against the globular head and/or the stalk region of HA.^6,7^

There is growing evidence that NA can provide relatively broad protection within a given NA subtype of influenza, yet, NA immunity is largely underexploited in currently licensed influenza vaccines. NA-specific immunity primarily depends on antibodies that have NA inhibition activity. For example, several NA-specific antibodies have been isolated that are capable of protecting against challenge with influenza A viruses from the same subtype, against multiple influenza B virus strains or, occasionally, even against multiple influenza A subtypes.^8–11^ Vaccination studies based on NA have shown varying degrees of success (reviewed in Ref. ^12^), where the best cross-protection is seen with strains that share a high sequence homology. Wohlbold et al.^13^ demonstrated that vaccination of mice with recombinant soluble tetrameric NAs derived from A/PR8/34 N1, A/Hong Kong/68 N2 or B/Yamagata/88 provided protection against challenge with a number of influenza viruses within the same subtype, even against strains that shared only 85% sequence homology with the recombinant NA vaccine. However, a high vaccine dose was required to induce cross-protective NA-inhibiting Antibodies. Furthermore, when mice were challenged with a high dose of heterologous virus the protection was diminished.^13^ Nevertheless, these studies highlight that cross-protective B-cell epitopes do exist within NA.

Here, we aimed to enhance the response directed towards conserved epitopes within the viral N1 subtype. We used a ‘cluster-based’ consensus (CBC) design approach to combine NA amino acid sequences covering three different sequence NA spaces: (i) H1N1 2009 pandemic and Swine H1N1 (NA5200), (ii) historic seasonal H1N1 (NA7900) and (iii) H1N1 viruses ranging from 1933 to the present (NA9100). Recombinantly produced consensus NAs induced antibodies that inhibited the NA activity of a broader number of N1 strains compared with wild-type NAs. Furthermore, we demonstrate that the CBC NA designs can protect mice from a broad range of H1N1 influenza viruses, even when natural NAs cannot.

## Results

### Design of H1N1 CBC NA genes

**Fig. 1:**
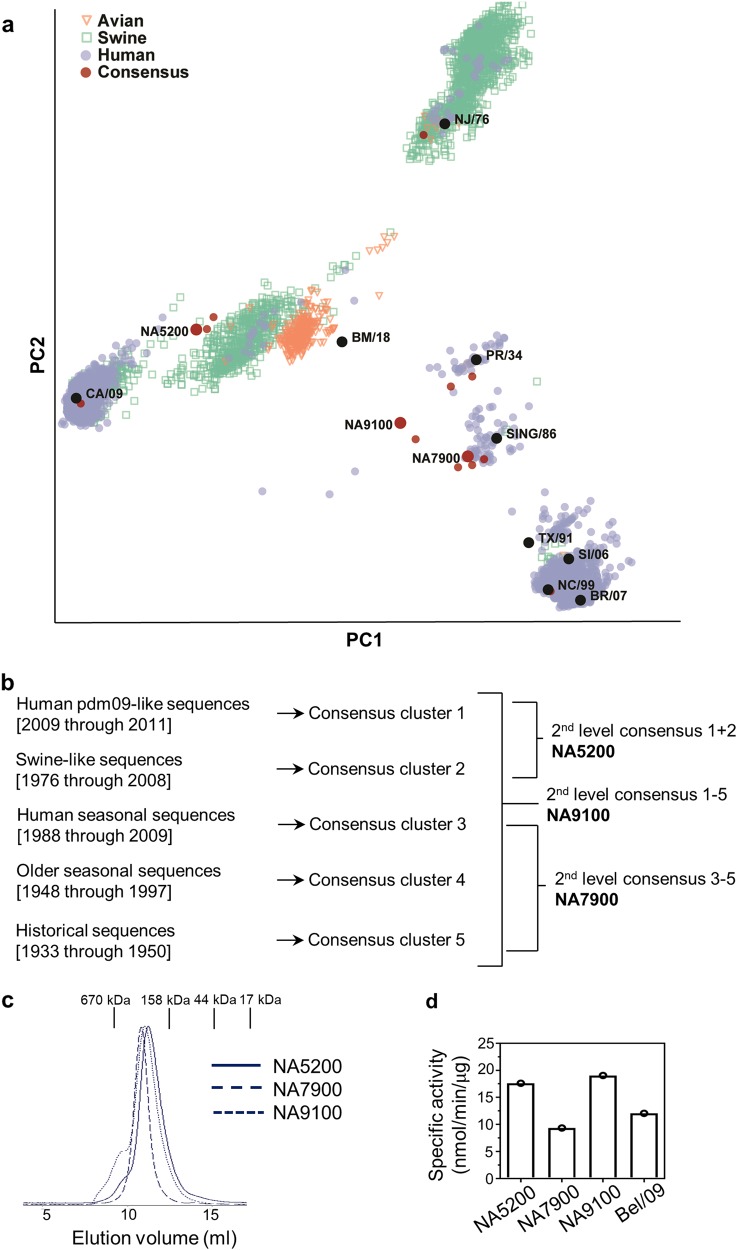
CBC design and production of recombinant tetrameric proteins. **a** Visualization of the first two dimensions from a multidimensional scaling analysis of the pairwise identity matrix of influenza A subtype H1N1 neuraminidase sequences. Host species represented by colour; Swine (green), Avian (orange), Human (purple). Consensus designs are highlighted in red. **b** Conceptual overview of CBC NA designs. **c, d** Purification profile and specific enzymatic activity of recombinant NAs. Soluble NA5200, NA7900 and NA9100 NAs were produced recombinantly in HEK 293 T cells. Supernatant was first affinity purified followed by size exclusion chromatography. **c** UV absorption profile after size exclusion chromatography for tetrameric soluble NAs. Vertical lines on top of the graph represents standard of known protein sizes. **d** Recombinant proteins, along with Bel/09 wild-type NA, were tested for enzymatic activity by MUNANA assay. Specific activity was calculated from a standard curve of 4-methylumbelliferone

The pairwise identity matrix for a non-redundant set of 1796, full-length, human N1 NA protein sequences were used as the input to partition sequence space by classical multidimensional scaling (MDS). Five clusters of similar protein sequences were defined representing historical seasonal-like sequences (1933–1950; 1948–1997; 1998–2009), swine-like sequences (1976–2008) and pandemic-like sequences (2009–2011) (Fig. 1a). Cluster-based consensus (CBC) NA sequences were generated by the combination of (i) swine-like and pandemic-like sequences yielding NA5200, (ii) historical seasonal-like sequence clusters to yield NA7900 and (iii) all five sequence clusters to yield NA9100, as depicted in Fig. 1b.

### Characterization of CBC recombinant soluble tetrameric NAs

As the NA proteins were designed according to criteria different from natural evolutionary processes, it was important to confirm correct folding and maintenance of epitope integrity. We tested NA enzymatic activity as a proxy for structural integrity, since it has been shown that while NA inhibiting antibodies can be induced by immunization with NA antigen that is enzymatically inhibited by the addition of zanamivir,^14^ the NA is still required to be in its native tetrameric form.^15^ We therefore produced and purified the computationally designed NA5200, NA7900 and NA9100 NA constructs as soluble tetrameric proteins (rNAs) in a mammalian expression system. Soluble tetrameric NA derived from H1N1pdm09 was produced as well and served as a naturally occurring control.^16^ Size exclusion chromatography analysis revealed a dominant peak for recombinant NA5200, NA7900 and NA9100 with a retention time that corresponded to the predicted molecular weight of a soluble tetrameric NA (Fig. 1c). For the NA5200 and NA9100 recombinant proteins, minor peaks were observed, which eluted faster from the size-exclusion column than the dominant peaks. These fractions likely corresponded to aggregate forms of NA, and were discarded. All three recombinant tetrameric NAs were enzymatically active as determined by the release of 4-methylumbelliferone from a small molecule sialic acid conjugate precursor. The specific activity of the three CBC recombinant tetrameric NAs was similar to or slightly higher than that of 2009 H1N1pdm-derived soluble tetrameric NA (Fig. 1d). Next, the potential N-glycosylation sites in the head domain of the CBC NAs were compared with those in relevant N1s. NA5200, NA7900 and NA9100 only carried three potential N-glycosylation sites in the head domain, which are found 100% conserved in nearly all N1 NAs.^17^ These are located at amino acid positions 88, 146 and 235 (Supplementary Table 1).

### CBC NAs elicit broad NA-inhibiting antibodies

**Fig. 2:**
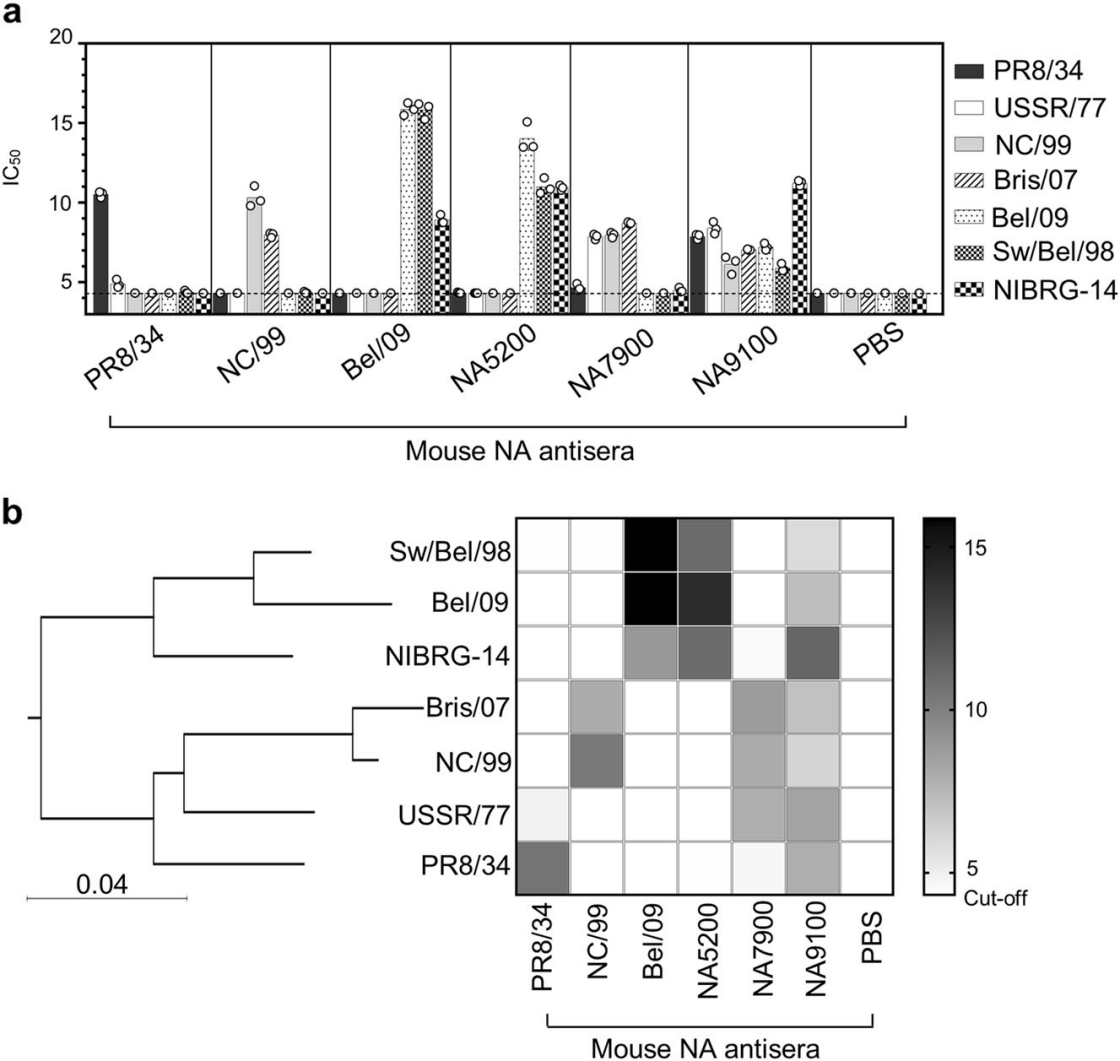
Anti-sera raised to CBC-designed NAs mediates NI against a broader range of influenza A viruses (IAV) than WT NA sera. Mice were vaccinated twice with 1 µg of PR8/34 rNA, NC/99 rNA, Bel/09 rNA, NA5200, NA7900, NA9100 or PBS alone, 3-weeks apart via the subcutaneous route in the presence of SAS. Three weeks following the boost anti-sera was collected and tested for their ability to inhibit the NA activity of a panel of N1 viruses. Increasing twofold serial dilutions of heat-inactivated sera raised to rNAs were mixed with a panel of N1 viruses and NA activity was determined at 18 h on fetuin coated plates by ELLA as described in Materials and Methods. Data shows IC_50_ values as determined by non-linear regression analysis and plotted as log_2_ values of the 1:*x* dilution resulting in 50% NA inhibition. **a** Data are reported as the mean of the experiment performed in triplicate and is representative of 2 independent experiments. The dotted line on the y axis represents the highest concentration of the sera tested, 4.3 (i.e., log_2_ of 1:20) and the cut-off of the assay. **b** Data represented as a heat map of IC_50_ values next to a phylogeny tree of NAs clustered by amino acid sequence of the NA head region

It is known from previous studies that protection by vaccination with NA largely depends on the induction of antibodies that can mediate neuraminidase inhibition (NI).^13^ Therefore, as an initial step to examine the potential breadth of the antibody response directed against the CBC NA designs, heat-inactivated sera raised against these molecules in mice were compared with sera from mice that had been immunized with three wild-type (WT) NAs for their capacity to mediate NI against a panel of human H1N1 viruses. Additionally, the H5N1 strain NIBRG-14 (a 6:2 reverse genetics reassortment containing HA, with the polybasic cleavage site deleted, and NA from A/Vietnam/1194/2004) and a swine H1N1 influenza isolate were included in the panel, as these strains represent potential pandemic-causing viruses. An IC_50_ titre (1:*x* dilution resulting in 50% NA inhibition) above a log_2_ value of 4.3 (i.e., 20-fold dilution; the lowest dilution of sera tested) was considered as a positive response. As observed with previous studies,^13^ immune sera raised against natural NAs possessed some cross-reactivity (Fig. 2). PR8/34 NA anti-sera mediated NI against PR8/34, and to a lesser extent against USSR/77 and Sw/Bel/98 (on average just above the cut-off point). Likewise, anti-sera induced by vaccination with purified recombinant tetrameric NA derived from NC/99 reacted with itself and Bris/07. Bel/09 NA anti-sera mediated the strongest NI titres against itself and Sw/Bel/98, followed by NIBRG-14. For the CBC NAs, the design strategy of the NA, for the most, dictated the panel of reactivity. Anti-NA5200 (H1N1 pdm09 and swine-like) anti-sera strongly inhibited the NA activity of Bel/09, Sw/Bel/98 and NIBRG-14. Anti-NA7900 (historical seasonal H1N1-like) immune serum mediated NI against all four seasonal H1N1 viruses tested (PR8/34, USSR/77, NC/99 and Bris/07) and NIBRG-14, displaying a broader NI span than anti-serum raised against NC/99 rNA (Fig. 2a,b). Finally, anti-NA9100 (H1N1 viruses 1933–2009) NA serum showed substantial NI against all N1 viruses tested.

Next, the percent sequence identity shared between the CBC NAs or wild-type (WT) NAs and the N1 viruses used in the ELLA was determined, considering only amino acids from 75 onwards, as the NA designs lack the native NA stalk sequence. NA9100 shared the highest percent identity with the N1 viruses tested, followed by NA7900, then NA5200. In comparison, the identity of WT NAs to other N1 NA varied greatly. In general, a higher degree of sequence identity between the WT viruses and the CBC-designed NAs, correlated with greater breadth of coverage in the NI (as shown in bold, Supplementary Table 2). Interestingly, although NC/99 NA shared 92% identity with USSR/77 there was no NI detected, even though the majority of the CBC NAs displayed NI at a ≥88% identity. This approach of percent sequence identity, however, does not take into account that some antigenic or conserved epitopes are discontinuous and bridge multiple regions of the monomer and even the tetrameric structure.^11,18^ Taken together, while the WT NAs do induce a degree of cross-reactivity, by using the consensus-based approach the breadth of cross-reactivity can be significantly enhanced, likely by targeting conserved epitopes within the N1 NA or combining epitope regions from multiple NA sequences into one molecule.

### Vaccination with CBC NAs provide protection against influenza A viruses in mice

**Fig. 3:**
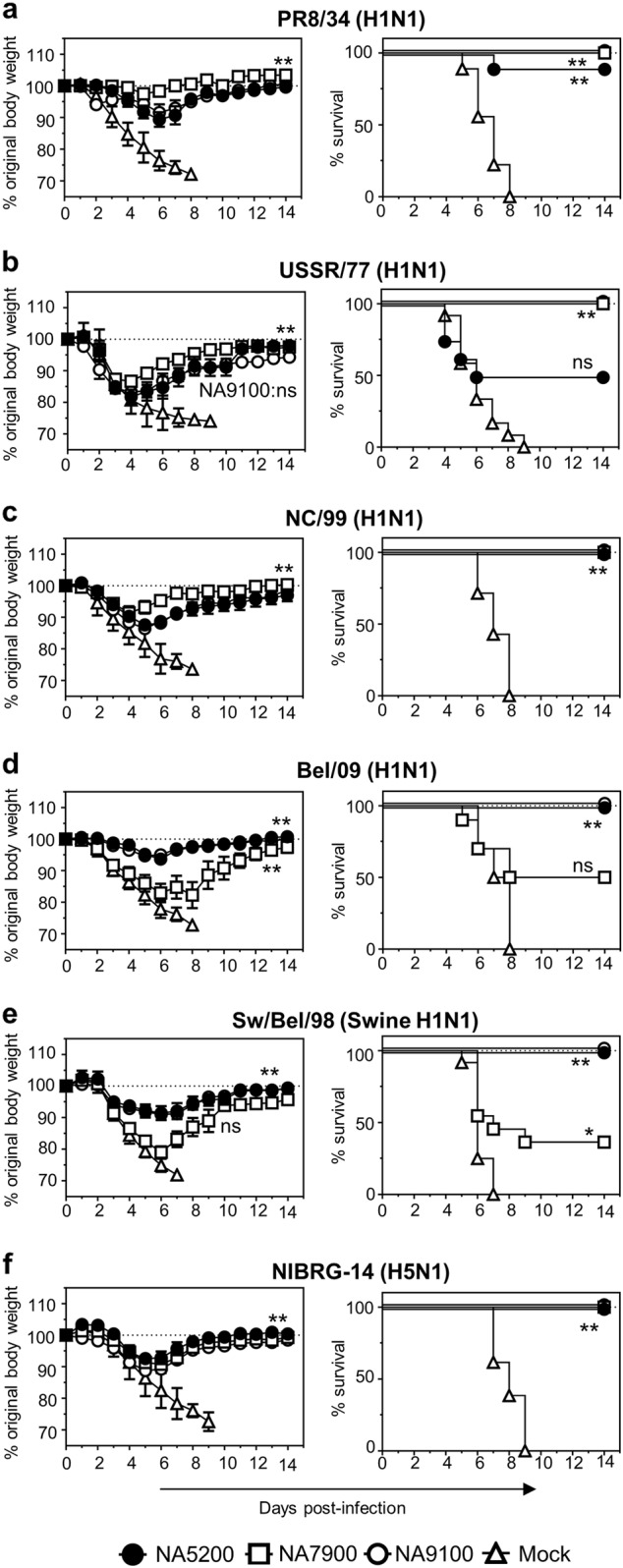
Vaccination with CBC NAs protects mice against a lethal infection with influenza A viruses. Mice were primed and boosted at a 3-week interval via the subcutaneous route with 1 µg of NA5200 (black circles), NA7900 (white squares) or NA9100 (white circles) rNA in SAS or mock vaccinated (white triangles). Following vaccination, mice were infected with 5 LD_50_ of either **a** PR8/34, **b** USSR/77, **c** NC/99, **d** Bel/09, **e** Sw/Bel/98 or **f** the H5N1 strain NIBRG-14 intranasally and monitored for weight loss (left panels) and mortality (right panels). When a mouse had lost ≥25% of its original body mass the animal was culled. Weight loss data are shown as the mean percentage (±SEM) of original body weight over time (*n* = 8–10), survival data are shown as the percentage of survival over time (*n* = 8–10). The data shown are pooled from two independent experiments. Weight loss was examined by two-way ANOVA, main column effects, and survival proportions were assessed using a two-tailed, log-rank (Mantel Cox) test. *P < 0.05, **P < 0.01 in comparison to mock-vaccinated mice

Next, the protective capability of the CBC NAs was examined in the mouse model. Mice were primed and boosted with NA5200, NA7900 or NA9100 rNA with adjuvant. Mice that had been mock-vaccinated with buffer only plus adjuvant were included as controls. Three weeks following the boost, mice were challenged with 5 LD_50_ of either PR8/34, USSR/77, NC/99, Bel/09, Sw/Bel/98 or NIBRG-14 and assessed over 14 days for body weight change and survival (Fig. 3). All challenged mice that received adjuvant alone succumbed to the infection by day 9 (Fig. 3). Vaccination with NA5200 significantly protected mice from weight loss and death, due to infection, with all N1 viruses tested, except USSR/77, compared with the controls. NA5200-vaccinated mice challenged with USSR/77 displayed transient weight loss over time and were only partially protected against mortality (Fig. 3b). NA7900 vaccination significantly protected mice from challenge with PR8/34, USSR/77, NC/99 or NIBRG-14, compared with mock-vaccinated animals, but only partially against Bel/09 and Sw/Bel/98. NA9100 showed a broad range of protection, significantly protecting mice from weight loss and death when infected with PR8/34, NC/99, Bel/09, Sw/Bel/98 and NIBRG-14. Mice infected with USSR/77 displayed no significant difference compared with mock-vaccinated mice, when considering main columns effects of a two-way ANOVA (*P*-value = 0.08). However, on days 6 through 9, there was a significant difference in weight loss compared with mock-vaccinated mice (*p* < 0.01, two-way ANOVA) and 100% of the mice survived the infection (Fig. 3b). Taking these results together, we can conclude that NA5200 and NA9100 rNAs provide broad protection potentially spanning a long time frame (approximately 80 years) and corresponding antigenic space.

**Fig. 4:**
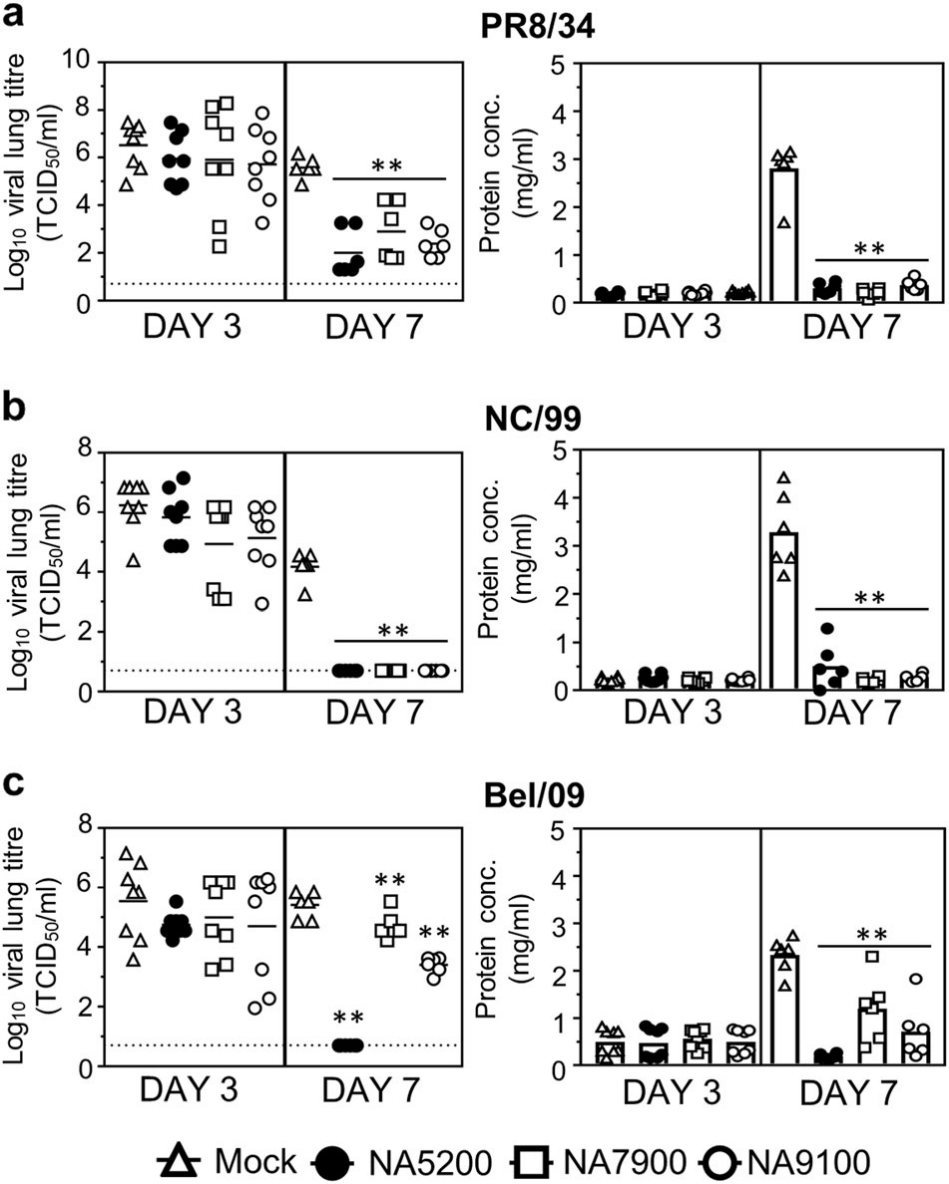
Protection mediated by CBC NAs results in a decrease of lung viral titres and inflammation within the lung milieu. Mice were primed and boosted with a 3-week interval via the subcutaneous route with 1 µg of NA5200, NA7900 or NA9100 rNA in SAS or mock-vaccinated with PBS in SAS. Following vaccination, mice were infected with 5 LD_50_ of either **a** PR8/34, **b** NC/99 or **c** Bel/09 intranasally. On day 3 or day 7 post-infection BAL fluids and lungs were taken and lung homogenates were assessed for viral titres by TCID_50_ (left panels). Cell-free BAL fluids were investigated for total levels of protein (right hand panels). The dashed line, in viral titre graphs, indicates the cut-off limit of the TCID_50_ assay (0.7). For protein concentrations; bars represent the mean. Data are pooled from two independent experiments. Significance was assessed using one-way ANOVA. *P < 0.05, **P < 0.01 in comparison to mock-vaccinated mice

Previous studies have shown that viral loads within the lung are decreased when anti-NA immunity is induced.^19^ Therefore, in separate experiments, we assessed if vaccination with NA5200, NA7900 or NA9100 could also reduce viral lung loads. Accordingly, mice were primed and boosted with either NA5200, NA7900 or NA9100 CBC NA and infected with 5 LD_50_ of PR8/34, NC/99 or Bel/09, 3 weeks following the boost (Fig. 4). On day 3 and day 7 post-infection, lungs were collected and viral loads examined. On day 3, viral loads did not show any significant reduction in the NA-vaccinated groups compared with mock-treated animals. By day 7 after challenge, however, all mice that had been vaccinated with consensus rNAs had significantly lower titres than the mock-vaccinated mice. Since vascular leakage and pulmonary oedema are indicative of a severe influenza infection, we also investigated the possible benefit of NA vaccination for these parameters based on the total protein content within cell-free Broncoalveolar lavage (BAL) fluids.^20,21^ Mice vaccinated with NA5200, NA7900 or NA9100 rNA and infected with either PR8/34, NC/99 and Bel/09 displayed no significant difference in the protein levels in BAL fluids isolated on day 3 after challenge, but had significantly less total protein on day 7 post infection compared with control mice (Fig. 4a–c, right panels).

### Passive transfer of CBC NA anti-sera protects against lethal challenge with influenza A virus

**Fig. 5:**
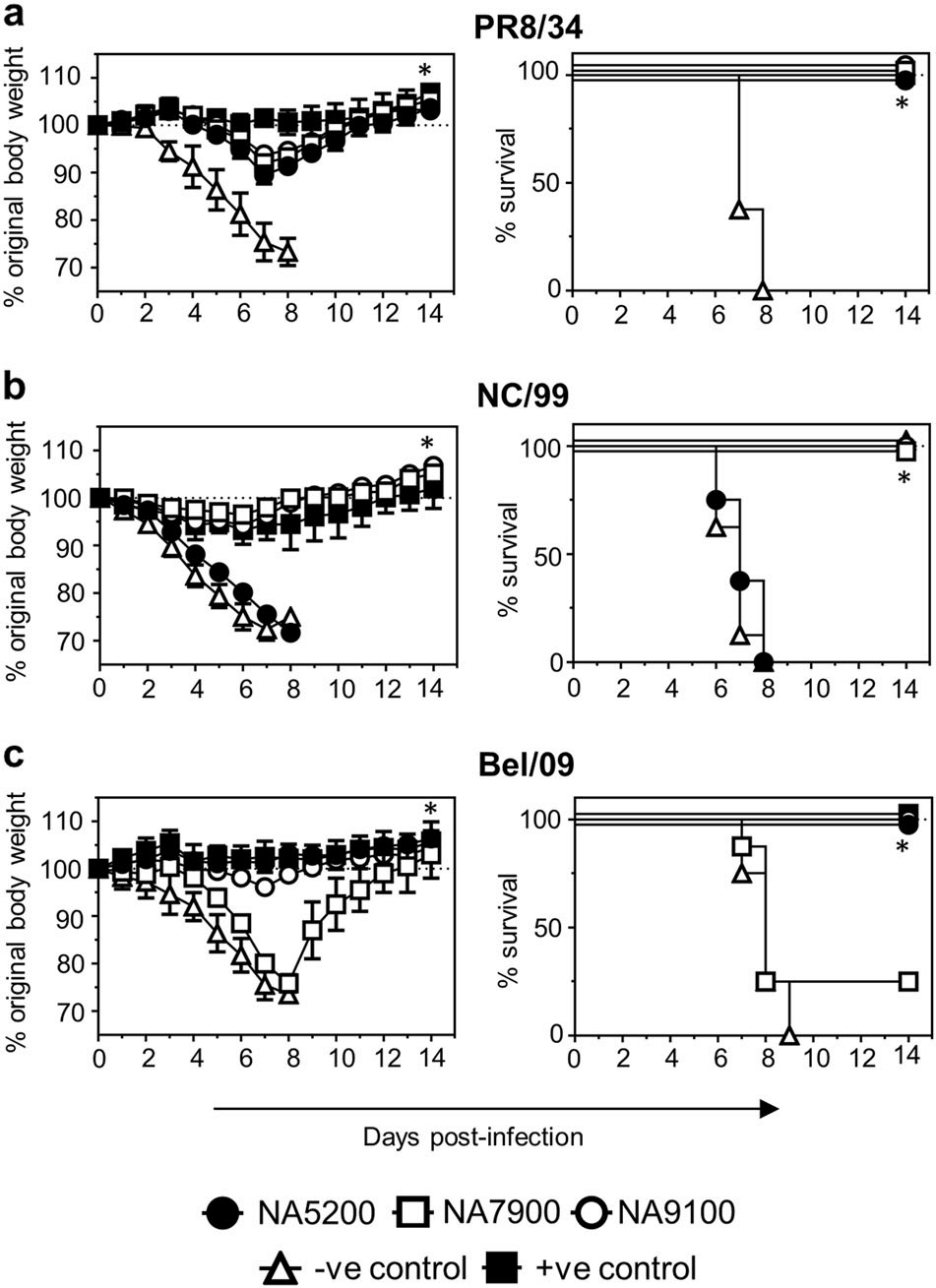
Antibodies play a major role in the protection provided by vaccination with CBC NAs. Anti-sera raised to CBC rNAs, WT PR8/34 rNA, WT NC/99 rNA, WT Bel/09 rNA (same as used in Fig. 2) were assessed for their ability to control influenza virus infection via passive transfer. Mice were treated intranasally with 20 µl of heat-inactivated anti-sera raised against (i) NA5200, (ii) NA7900, (iii) NA9100, (iv) PBS (negative; -ve control) or (v) respective homologous rNA positive (+ve) control. Twenty-four hrs later they were challenged with 2 LD_50_ of **a** PR8/34, **b** NC/99 or **c** Bel/09 via the intranasal route and monitored over 14 days for weight loss (left panels) and survival (right panels). If mice had lost ≥25% of their original body weight they were euthanized. Weight loss data displays the mean percentage (±SEM) of original body weight over time (*n* = 8) and survival data is shown as the percentage of survival over time (*n* = 8). The data are pooled from two independent experiments. A two-way ANOVA was used to analyse weight loss over time, assessing main column effects, whilst a two-tailed, log-rank (Mantel Cox) test was used to assess survival. *P < 0.05 in comparison with negative control mice

The major correlate of protection induced by vaccination with rNA is the ability to induce NA inhibiting antibodies.^12^ To examine if antibodies were the major mediators of protection induced by vaccination with CBC NAs in this mouse model, heat-inactivated anti-sera raised separately against NA5200, NA7900, NA9100 or buffer alone (PBS) were passively transferred intranasally to mice 1 day prior to infection with 2 LD_50_ of either PR8/34, NC/99 or Bel/09 (Fig. 5). Anti-sera raised to individual WT rNAs were included as homologous-positive controls. Anti-sera were the same sera used previously in Fig. 2 to assess the broadening of NAI responses. In all viral challenges, the positive control anti-sera fully protected mice from weight loss and survival following a potentially lethal infection with the homologous virus. Although active vaccination with NA5200 rNA protected against PR8/34, NC/99 and Bel/09 infection, passive transfer of NA5200 anti-serum only provided significant protection against weight loss and mortality in mice infected with Bel/09 or PR8/34, and not with NC/99 (*p* < 0.01 two-way ANOVA or log-rank test) (Fig. 5). NA7900 rNA immune serum recipient mice were significantly protected against morbidity and mortality when infected with PR8/34 and NC/99, in comparison with the negative control group (*p* < 0.01 two-way ANOVA or log-rank test). Similar to the poor protection by NA7900 rNA active vaccination against Bel/09 challenge, protection against this challenge virus by passive transfer of NA7900 anti-serum was not significantly different from the negative control group. Finally, the passive transfer of NA9100 anti-serum provided significant protection against all three challenge viruses for both weight loss and survival (*p* < 0.01 two-way ANOVA or log-rank test). In all passive transfer experiments (Fig. 5), full protection against mortality correlated with the ability of the anti-sera to mediate NI (Fig. 2). As such, it can be concluded that antibodies play a major role in the protection induced by the CBC NAs.

### CBC NAs provide broader protection than WT NAs and increase the breadth of a split inactivated vaccine

**Fig. 6:**
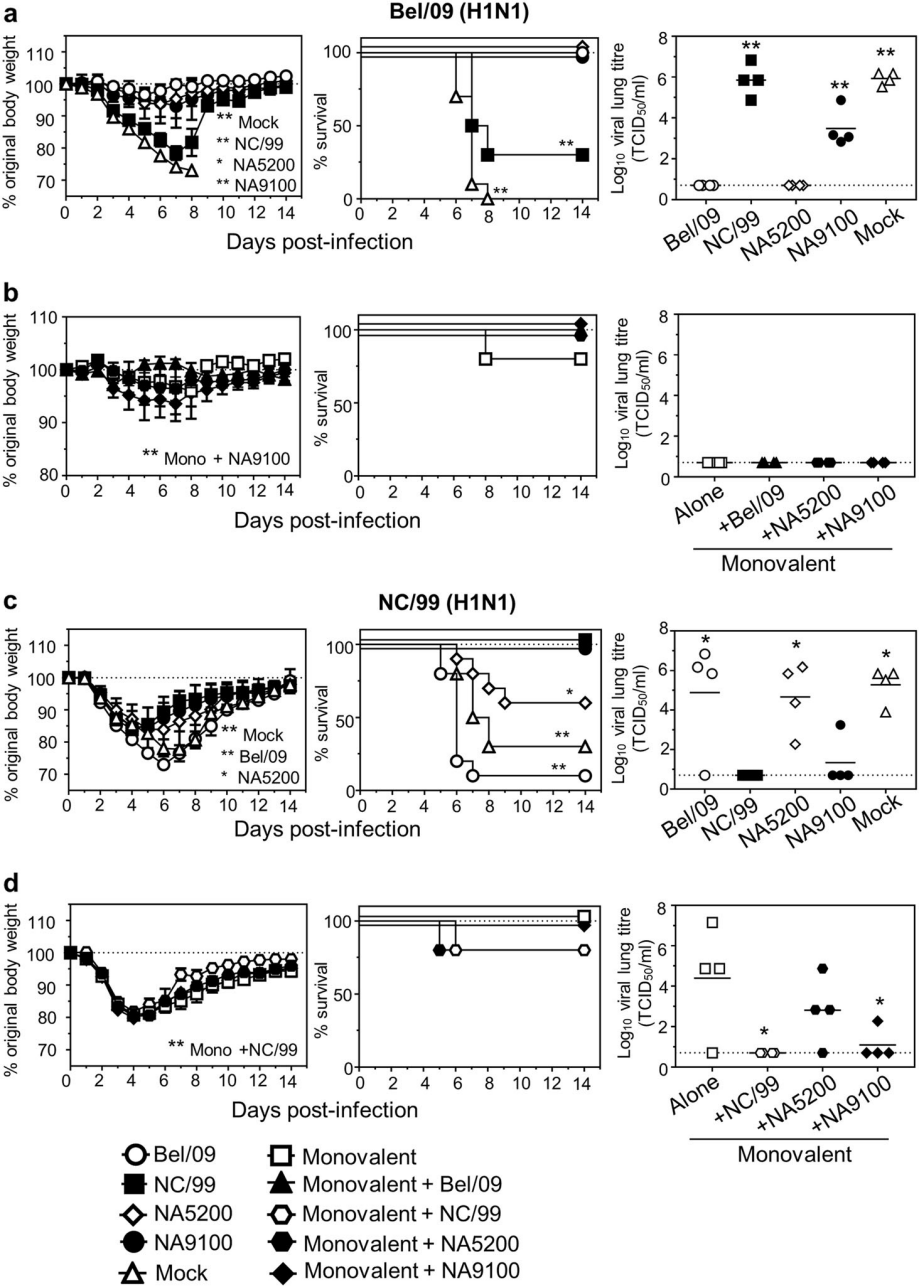
CBC NAs provide broader protection than WT NAs and provide increased breadth when added to monovalent inactivated vaccine. Mice were primed and boosted at a 3-week interval via the subcutaneous route, in the presence of SAS adjuvant, with either 1 µg of WT Bel/09 rNA, WT NC/99 rNA, CBC NAs NA5200, NA9100 or with 0.1 µg of monovalent pdm09 vaccine alone or in combination with 1 µg of WT Bel/09 rNA, WT NC/99 rNA, CBC NAs NA5200, NA9100. A group of mice was also mock vaccinated. Three weeks following the final boost, the mice were challenged with 5 LD_50_ of Bel/09 or NC/99 via the intranasal route. Mice were monitored for weight loss (left panels) and survival (middle panels) over 14 days. Mice were sacrificed if they lost ≥25% of their original body mass. On day 7 for Bel/09 infections and day 6 for NC/99 infections lung homogenates were obtained and assessed for viral load. Weight loss data represents the mean percentage (±SEM) of original body weight over time and survival data are shown as the percentage of survival over time (*n* = 9–10 for **a**, **c** pooled from two independent experiments and *n* = 5 for **b**, **d** from one experiment). Weight loss over time was analysed by two-way ANOVA, examining main column effects, and survival proportions were assessed using a two-tailed, log-rank (Mantel Cox) test. Significance was assessed using one-way ANOVA for viral titres. *P < 0.05, **P < 0.01 in comparison to homologous rNA-vaccinated mice for **a**, **c** or in comparison to monovalent alone vaccinated mice for **b**, **d**

To test if the CBC NAs could (i) offer broader protection in vivo compared with WT rNAs and (ii) increase upon the protection provided by a conventional split inactivated vaccine, mice were vaccinated with NA5200 and NA9100 or WT soluble recombinant NAs derived from Bel/09 and NC/99 alone or in combination with a monovalent H1N1 pdm09 vaccine. Subsequently, the mice were challenged with 5 LD_50_ of Bel/09 or NC/99. Mice vaccinated with NC/99 rNA were not protected from weight loss and mortality following Bel/09 challenge. Also following Bel/09 challenge, both NA5200 and NA9100-vaccinated mice displayed slightly more, yet significant, weight loss in comparison with homologous-vaccinated mice. However, 100% of these mice survived the infection (Fig. 6a, left and middle panels). Furthermore, although viral loads in the lungs on day 7 of NC/99-vaccinated mice were on average similar to mock-vaccinated mice, viral titres on day 7 of NA5200 and NA9100-vaccinated mice were significantly reduced compared with mock-vaccinated mice (*p* < 0.01, one-way ANOVA), and both homologous Bel/09 rNA and NA5200 out-performed NA9100 NA (Fig. 6a, right panels). A similar, but reversed pattern was observed for mice challenged with seasonal NC/99 when receiving the more ‘seasonal’-based NA9100 (Fig. 6c). In this case, Bel/09 rNA-vaccinated mice lost significantly more weight, displayed increased mortality and higher viral loads at day 6 post-infection compared with NC/99-vaccinated mice (Fig. 6c left and middle panels). Compared with the data presented in Fig. 3c, NA5200 did not protect NC/99 challenged mice to the same degree, but NA9100 was not significantly different in its ability to protect mice compared with homologous-vaccinated mice. In addition, Bel/09 rNA could also not protect mice from morbidity and mortality when infected with PR8/34 virus (data not shown), while all CBC tetNA induced protection (Fig. 3). Taken together, these data show that NA9100, in particular, shows broader protection than the WT NAs tested.

Mice vaccinated with monovalent H1N1 pdm09 split vaccine (alone or in combination with CBC rNAs) and challenged with Bel/09 showed little signs of weight loss, almost no mortality and no signs of virus in the lungs by day 7 post-infection (Fig. 6b). These results are as expected after immunization with the monovalent vaccine, especially in combination with an adjuvant, and is known to provide protection against homologous viruses, with little room for improvement within this model.^22^ Protection provided by the monovalent vaccine is most likely attributable to HA content, as the monovalent H1N1 pdm09 vaccine has been shown to contain a low amount of intact NA.^23^ When mice were challenged with NC/99, there was also a degree of protection provided by the adjuvanted split vaccine alone compared to mock-vaccinated mice (Fig. 6c,d). Both NA5200 and NA9100 showed no additive effect on weight loss and survival in comparison with monovalent only. However, the addition of NC/99 or NA9100 NAs to the monovalent split vaccine significantly reduced viral titres in the lungs compared to monovalent alone (Fig. 6d). As such, while we only saw a modest increase in protection compared to monovalent alone vaccine, these data highlight the ability of rNA to augment protection when combined with the traditional vaccine approach.

### NA5200 and NA9100 NA anti-sera inhibits the NA activity of a A(H1N1)pdm09 HA variant

For the 2017 Southern Hemisphere influenza season, the World Health Organization (WHO) recommended to replace the A(H1N1)pdm09-like virus in the seasonal influenza vaccine with a A/Michigan/45/2015-like strain,^24^ as it was evident this variant possessed a change within the HA that resulted in increased infection rates in middle-aged adults.^25,26^ It was therefore important to test if the CBC designs could also mediate NI against this variant, as its sequence was not included in the original design strategy. Initially, we investigated the HA antigenic difference using hemagglutination inhibition (HI), between Bel/09 and the A/Michigan/45/2015-like virus A/Singapore/GP1908/2015 (Sing/15). We observed a twofold difference in the ability of anti-sera raised in mice against the monovalent split A(H1N1)pdm09 vaccine to mediate HI against Bel/09 and Sing/15 (1280 HAU vs 640 HAU, data not shown), which is in agreement with previous studies where ferret reference sera did not indicate evidence of significant antigenic drift.^25,26^ Next, the ability of anti-sera raised against the CBC rNAs, or Bel/09 rNA, to mediate NI against Bel/09 and Sing/15 was tested. Whereas Bel/09 immune serum had a significantly lower NI titre against the drift variant Sing/15 as compared with its homologous titre (*p* < 0.05, one-way ANOVA), anti-sera raised to NA5200 and NA9100 mediated NI against both Sing/15 and Bel/09 equally well albeit at lower levels than WT Bel/09 anti-NA sera (Supplementary Fig. 1A/B). When considering amino acid identity from sequence position 75 onwards, Sing/15 shared 91%, 87%, 89 and 97% identity to NA5200, NA7900, NA9100 and Bel/09, respectively (Supplementary Table 2). Whether the NA of Bel/09 and Sing/15 are antigenically different is not known and if the significant difference between the ability of anti-Bel/09 sera to mediate NI between Sing/15 and Bel/09 reflects a difference antigenically is also not known. Of importance, though, is that the CBC NAs displayed the same level of inhibition against a strain that was not included in the original design of the proteins but is considered drifted with regards to HA and is now the new vaccine strain.

## Discussion

The target set by the WHO by 2020 is to have “at least one vaccine providing broad spectrum protection against influenza A licensed”.^27^ Many recent advances in the field have been aimed at addressing this target and several approaches are in preclinical or clinical trial phases (reviewed in Ref. ^28^). Previous studies have also used a consensus-based approach for NA constructs used in DNA vaccination studies. The authors designed a consensus-based sequence of NA based on H1N1 and H5N1 viruses that were available before the H1N1pdm09 outbreak and partial protection was observed when mice were challenged with a lethal dose (100 LD_50_) of the H5N1 virus A/Hanoi/05/2005. Cross-reactive responses induced by the consensus NA, however, were not examined.^29,30^ In the present study, we describe a consensus-based approach that broadens the antibody response directed towards influenza NA, leading to coverage of N1 strains from 1934 until recent times, and highlighting the potential of NA designs as vaccine candidates capable of providing broad intrasubtypic (with respect to NA) protection against influenza. Our data further support existing literature that NA needs to be part of a broadly cross-protective/universal influenza vaccine strategy.

Importantly, we demonstrated that considerable NI could be induced against a variant of the H1N1pdm09 virus, which was not included in the design of the consensus NAs. This variant had not been identified in the conventional ferret reference sera HAI assay as an antigenic drift variant, however considerable HAI differences in the reactivity of human sera from adults born between 1965 and 1975 against this H1N1pdm09 variant, have been observed. For instance, the authors of some studies hypothesized that pre-immunity in these individuals had led to a difference in the response mediated against the drift variant, indicative of original antigenic sin.^25,26^ Although, the degree of antigenic difference between the NA of A(H1N1)pdm09 isolates and A/Michigan/45/2015-like viruses are incompletely known, our NI results suggest it is possible to induce anti-NA responses using both WT and CBC-designed rNAs that can bridge to strains that are antigenically drifted according to their HA variants. It is noteworthy that the magnitude of NI directed to Bel/09 and Sing/15 by the CBCs rNA is lower than what is induced by Bel/09 rNA. This is also evident in Fig. 2, where WT NAs induce higher homologous NI titres than CBC-designed NAs and in Fig. 6 where in some cases the homologous NA outperforms the CBC-designed NA. As such it seems that the increased breadth and a more 'antigenically balanced' response comes at a cost of losing high homologous titres. Importantly, at least in the mouse model of infection, NI titres induced by CBC NAs could protect against weight loss and lethality. Future clinical studies are needed to define levels of NI that correlate to protection or reduced infection in humans which could be used to assess the protective capabilities of the CBC designs.

By passive transfer of anti-sera, we demonstrated that antibodies capable of NI were the main correlate of protection provided by the CBC NAs. However, in some cases (protection of NC/99- infected mice by NA5200 rNA in the absence of detectable NI), it was clear that another mechanism other than NI was involved. Several studies have identified epitopes for CD4+ and CD8+ T cells within the NA.^31–33^ We speculate that T cells could play a role in the protection of mice, however, further studies are required to confirm this. It is also possible that antibody-dependent effector mechanisms, for example, antibody-dependent cellular cytotoxicity (ADCC) or antibody-dependent cellular phagocytosis (ADCP) could contribute to protection. Previous studies have proposed that anti-NA antibodies lacking detectable NI rely on the engagement of Fcγ-receptors to protect in the mouse model.^34^ Further, antibodies directed towards NA can mediate in vitro ADCC^10,35^ albeit in some cases weakly.^36^ The contribution of ADCC and ADCP mediated by anti-NA antibodies to protection in mice is, however, largely ill-defined.

Currently 9 NA subtypes with enzymatic activity have been identified.^37^ They can be grouped phylogenetically into group 1 containing N1, N4, N5 and N8 and group 2 containing N2, N3, N6, N7 and N9.^38^ Previously, Wohlbold et al. have described that limited or no heterosubtypic immunity is induced by wild-type recombinant N3, N5, N6, N7, N8 and N9 against a H1N1 virus (PR8/34) and a H3N2 virus (X-31; A/Hong Kong/1968).^13^ As the CBC-designed NAs induced a broader profile of NI against heterologous strains than WT NAs within the N1 subtype, it would be interesting to investigate if the CBCs can provide heterosubtypic immunity among group 1 viruses, which share greater genetic similarity. Vaccine effectiveness of the TIV strategy against H3N2 is lower than that of H1N1 or influenza B viruses.^39^ Moreover, H3N2 is the leading cause for severe influenza disease^40^ and displays faster genetic and antigenic drift than H1N1 viruses.^41^ As the antigenic landscape to cover is far greater for human H3N2 viruses than for H1N1 viruses,^41^ it remains to be seen whether a similar approach of CBC-designed NA would also induce broad protective immunity against H3N2 viruses. Influenza B virus circulates as two distinct lineages, Victoria and Yamagata, based on HA antigenicity, whereas the NA contains several cross-reactive epitopes.^10^ Accordingly, vaccination of mice with recombinant NA derived from B/Yamagata/1988 was able to protect against two Victoria lineage strains; B/Victoria/1987 and B/Malaysia/2004.^13^ High NI titres were seen against B/Yamagata/1988 and B/Victoria/1987 but were limited against B/Malaysia/2004. Therefore, we speculate that a CBC-based approach could boost the already existing cross-protective responses within the influenza B lineages, leading to a more robust vaccine. The rationale for this approach is strengthened by the fact that since the mid 1990’s both influenza B lineages carry the NA from the Yamagata lineage.^42^

Vaccination with CBC-designed NAs is infection-permissive in the mouse model and only moderately reduces viral titres within the lung milieu. In contrast, the aim of seasonal TIV and QIV vaccines is to provide sterilizing immunity. Previous studies from our group show the benefit of an infection-permissive preclinical vaccine strategy, which allows for the development of cross-protective CD8+ cellular-mediated responses directed towards the internal nucleoprotein.^16^ In the present study, some mouse groups vaccinated with the CBC-designed NAs are 100% protected against lethality, although some mice displayed significant signs of morbidity. Antibodies directed to NA in humans have been shown to be correlated to protection independent from HA-mediated immunity. Furthermore, reduction in clinical signs of disease and viral titres correlated better to NI titres than to HI titres, and as NI titres increased there was less likelihood of vaccine failure.^43^ As such, we would argue that while NA-mediated immunity may be infection-permissive in mice, in humans it remains an important correlate of protection. In sum, we have applied a cluster-based consensus sequence-based approach to broaden the antibody response against the influenza N1 subtype and highlight the possibility of this technique to provide broad protection against influenza viruses. These data also support the inclusion of NA in any broadly cross-protective design strategy.

## Methods

### Viruses

Type A influenza viruses (IAV) used in this study were the mouse-adapted H1N1 strains A/USSR/90/1977 (USSR/77), A/New Caledonia/20/1999 (NC/99), A/Brisbane/59/2007 (Bris/07) and A/Belgium/145-MA/2009 (Bel/09).^16^ A/Puerto Rico/8/34 (PR8/34) and the H5N1 virus NIBRG-14, a 2:6 reverse genetics-derived reassortant expressing the NA and HA (with the polybasic motif removed) segments of A/Vietnam/1194/2004 and the other six genes segments from PR8/34 (obtained from the UK national Institute for Biological Standards and Control) were also used. The A/Swine/Belgium/1/98 strain, a kind gift from Kristien van Reeth, was also mouse adapted by consecutive passage in mouse lungs.^3^ The A(H1N1)pdm09 drift variant, A/Singapore/GP1908/2015 (Sing/15), was obtained from the National Influenza Centre, the Netherlands.

IAVs were propagated in MDCK cells in serum-free media in the presence of TPCK-trypsin (Sigma-Aldrich), and the median tissue culture infective dose (TCID_50_) and median lethal dose (LD_50_) of viruses were calculated by the Reed and Munch method. Standard H1N1 numbering is used throughout this study.

### Design and production of recombinant consensus NA proteins

Influenza A NA protein sequences (subtype H1N1) from 1933 through to 2011 were downloaded from the Influenza Virus Resource at the National Center for Biotechnology Information.^44^ A non-redundant set of 1796 full-length, human-host, NA protein sequences was identified for analysis and consensus-sequence generation. The non-redundant set of protein sequences was aligned with MAFFT v7^45^ and the all versus all pairwise identity matrix was generated and classical multidimensional scaling performed using custom scripts in R and python. Five clusters of similar protein sequences were defined representing seasonal-like sequences (1933–1950; 1948–1997; 1998–2009), swine-like sequences (1976–2008) and pandemic-like sequences (2009–2011). Cluster-based consensus (CBC) NA sequences were generated by the combination of (i) swine-like and pandemic-like sequences yielding NA5200, (ii) seasonal-like sequence clusters to yield NA7900 and (iii) all five sequences clusters to yield NA9100 (Fig. 1a, b). The 3D structures of sequences generated by the consensus method were modelled using the Rosetta Molecular Modelling package.^46^ Where residue positions could not be assigned unambiguously using the consensus generation algorithm, the amino acid resulting in a structure with the lowest calculated potential energy was selected, since it was presumed to be more stable and therefore likely to be expressed. The sequences for the three CBC NAs have been reported in US patent filing 62649002. The amino acid sequences are shown in Supplementary Table 1. Prediction of possible N-glycosylation sites was performed using the NetNGlyc 1.0 server.

Recombinant tetrameric NAs (rNA) were produced essentially as described previously for Bel/09 rNA.^16^ In brief, the stalk of the NA monomers was replaced by a helical peptide derived from *Tetrabrachion* which self-assembles into a tetrameric coiled-coil, thereby stabilizing the tertiary and quaternary structure of the NA enzyme domain. Secretion was facilitated by an N-terminal CD5-derived secretion signal, and a Strep-tag was cloned between the secretion signal and the coiled-coil for purification.^47^ The expression constructs encoding the sequences of NA5200, NA7900 and NA9100 were ordered from GenScript. The NAs from wild-type viruses (PR8/34, NC/99, Bel/09) were isolated by RT-PCR of RNA isolated from MDCK cell-grown virus. All NA sequences were cloned into pEF1/V5 (ThermoFisher Scientific). rNA was affinity-trapped from the supernatant of transiently transfected HEK293T cells using a StrepTrap HP column and eluted with 2.5 mM desthiobiotin in phosphate buffered saline (PBS) followed by size exclusion chromatography in PBS using an AKTAexplorer purification system (GE Healthcare Life Sciences). Recombinant NA activity was determined by cleavage of 4-methylumbelliferone from 2′-(4-methylumbelliferyl)-α-D-N-acetylneuraminic acid (1 mM) by recombinant NAs in 200 mM NaAc (pH 6.5), 2 mM CaCl_2_ and 1% butanol at 37 °C. Release of 4-methylumbelliferone was measured every 2 min for 1 h (355 nm and 460 nm). One NA unit is defined as the amount of NA that releases 1 nmol of 4-methylumbelliferone per minute.

### NA inhibition (NI) assays

Anti-sera against the different NAs were tested for their ability to inhibit the activity of the viral NA using the standard enzyme-linked lectin assay (ELLA) basically as described in Couzens et al. ^48^ In brief, serial dilutions of heat-inactivated sera (starting dilution of 1:20) were incubated with IAV at a pre-determined concentration of virus to give 90% maximal NA activity, for 30 min at 37 °C in PBS supplemented with 10 mg/ml BSA, 1 mM CaCl_2_, 0.5 mM MgCl_2_ and 0.5% Tween20. Dilutions were added to PBS plus 0.1% Tween washed wells of 96-well MaxiSorp plates (Nunc), coated with fetuin (Sigma-Aldrich, 5 µg/ml) and incubated for 18 h at 37 °C. HRP-coupled peanut agglutinin (PNA, Sigma-Aldrich, 2.5 µg/ml) was used to detect galactose residues exposed after NA-mediated removal of sialic acid from fetuin. The 50% inhibition concentration (IC_50_) was calculated by non-linear regression analysis (GraphPad Prism). The IC_50_ is defined as the reciprocal of the serum dilution, expressed as log_2_, that results in 50% inhibition of NA activity. A cut-off value of an IC_50_ of 20 was set, the highest concentration of sera tested, any points above this were considered positive for NI.

### Mouse studies

Female BALB/C mice (Charles River) were housed in specific pathogen-free conditions with food and water ad libitum. All mouse experiments complied with national (Belgian Law 14/08/1986 and 22/12/20333, Belgian Royal Decree 06/04/2010) and European legislation (EU Directives 2010/63/EU, 86/609EEG) on animal regulations. The ethics committee of the Vlaams Instituut voor Biotechnologie (VIB), Ghent University, Faculty of Science (Eth. Com No. 2014–068 and 2016–059) approved all experiments.

Six-week-old mice were primed and then boosted subcutaneously after a 3-week interval with 1 µg of recombinant CBC NAs or control NAs or 0.1 µg HA of influenza A (H1N1) 2009 monovalent vaccine (sourced from BEI resources, NR-20083, for the prime and Sanofi Pasteur for the boost) alone or in combination with NA using the Sigma Adjuvant System (SAS), containing the immuno-stimulants monophosphoryl Lipid A and synthetic trehalose dicorynmycolate. Sera samples were taken by tail bleeding at 3 weeks after the prime and boost. In some cases, terminal bleeds were performed 3 weeks after the boost vaccination by retro-orbital bleeding to obtain a pool of sera, which was heat-inactivated to test in NI and passive transfer experiments. For active vaccination challenge experiments, mice were infected intranasally (i.n.) with 5 LD_50_ of influenza virus in a total volume of 50 µl, 3 weeks after boost vaccination under isoflurane anaesthesia. For passive transfer experiments, six-week-old mice were administered 20 µl of heat-inactivated immune sera 1 day prior to infection with 2 LD_50_ of influenza virus via the intranasal route. Mice were subsequently monitored for weight loss and survival and were euthanized if they lost ≥25% of their original body weight. In some experiments on days 3, 6 or 7, mice were killed by overdose of Natrium Pentobarbital (final concentration 3 mg/mouse), and bronchoalveolar lavages (BAL) were performed and lungs were excised. BAL were performed according to Van Hoecke et al.^49^ Total protein levels in cell-free BAL fluids were determined by Bradford protein dye using a standard curve of BSA. Lungs homogenates were prepared and clarified as previously described^50^ and viral titre assessed by TCID_50_ assay (see below).

### TCID_50_

Standard TCID_50_ assays were used to assess viral titres in the clarified lung homogenates. Confluent monolayers of MDCK cells in 96-well plates, cultured in DMEM plus 10% FCS, and supplemented with non-essential amino acids, 2 mM L-glutamine, 0.4 mM sodium-pyruvate, were washed in serum-free media and incubated with 10-fold dilutions of samples in serum-free DMEM containing 1 µg/ml of TPCK-treated trypsin. Virus was detected in the wells by agglutination of chicken red blood cells after 7 days post-infection, and values were calculated by the Reed and Muench method.

### Statistical analysis

When comparing three or more sets of values, the data were analysed by one-way ANOVA followed by post hoc analysis using Tukey’s multiple comparison test. For changes over time, a two-way ANOVA was used. A log-rank test assessed survival significance. *P*-values < 0.05 were considered significant. To assess correlation between different variables, Pearson correlation was applied. All analysis was performed using GraphPad Prism software.

## Electronic supplementary material

Supplementary tables 1 and 2

## Data Availability

The data that support the findings of this study are available from the corresponding authors upon request.

## Electronic supplementary material

**Supplementary information** accompanies the paper on the *npj Vaccines* website (10.1038/s41541-018-0093-1).
