# Supplementary material for: Broadened immunity against influenza by vaccination with computationally designed influenza virus N1 neuraminidase constructs
Source: NPJ Vaccines. 2018 Nov 29;3:55. doi: 10.1038/s41541-018-0093-1 (PMC6265323; doi:10.1038/s41541-018-0093-1)
Supplement: Supplementary file 1 — Supplementary tables 1 and 2 [file 41541_2018_93_MOESM1_ESM.pdf]

Supplementary Table 1. Amino acid sequences of CBC designed NAs and predicted N-glycosylation sites

CBC NA	Sequence
**NA5200**	MNPNQKIITI GSVCMTIGMA NLILQIGNII SIWISHSIQL GNQNQIETC**N QS**VITYE**NNT** WV**NQT**YV**NIS**<br>NTNFAAGQSV VSIKLAG**NSS** LCPISGWAIY SKDNSIRIGS KGDVFVIREP FISCSHLECR TFFLTQGALL<br>NDKHS**NGT**VK DRSPYRTLMS CPIGEAPSPY NSRFESVAWS ASACHDGMGW LTIGISGPDN GAVAVLKYNG<br>IITDTIKSWR NKILRTQESE CACV**NGS**CFT VMTDGPSDGQ ASYKIFKIEK GKIVKSVEMD APNYHYEECS<br>CYPDSGKVMC VCRDNWHGSN RPWVSFDQNL DYQIGYICSG IFGDNPRSND GTGSCGPVSS NGANGVKGFS<br>FRYGNGVWIG RTKSISSRKG FEMIWDPNGW TETDNSFSIK QDIVAITEWS GYSGSFVQHP ELTGMNCIRP<br>CFWVELIRGQ PKENTIWTSG SSISFCGVNS DTVGWSWPDG ADLPFTIDK
**NA7900**	MNPNQKIITI GSICMAIGII SLILQIGNII SIWVSHSIQT GSQ**NHT**GICN QRIITYE**NST** WV**NQT**YVNI**N**<br>**NT**NVVAGKDT TSVILAG**NSS** LCPIRGWAIY SKDNSIRIGS KGDVFVIREP FISCSHLECR TFFLTQGALL<br>NDKHS**NGT**VK DRSPYRALMS CPVGEAPSPY NSRFESVAWS ASACHDGMGW LTIGISGPDN GAVAVLKYNG<br>IITETIKSWR KNILRTQESE CVCV**NGS**CFT IMTDGPSDGL ASYKIFKIEK GKVTKSIELD APNSHYEECS<br>CYPDTGKVMC VCRDNWHGSN RPWVSFNQNL DYQIGYICSG VFGDNPRPKD GTGSCGPVTV DGANGVKGFS<br>YRYGNGVWIG RTKSNSSRKG FEMIWDPNGW TETDSSFLVK QDVVAITDWS GYSGSFVQHP ELTGLDCMRP<br>CFWVELIRGL PREDTIWTSG SSISFCGVNS DTVNWSWPDG AELPFTIDK
**NA9100**	MNPNQKIITI GSICMAIGII SLILQIGNII SIWVSHSIQT GSQ**NHT**GICN QRIITYE**NST** WV**NQT**YVNI**N**<br>**NT**NVVAGKDT TSVILAG**NSS** LCPIRGWAIY SKDNSIRIGS KGDVFVIREP FISCSHLECR TFFLTQGALL<br>NDKHS**NGT**VK DRSPYRTLMS CPVGEAPSPY NSRFESVAWS ASACHDGMGW LTIGISGPDN GAVAVLKYNG<br>IITDTIKSWR NNILRTQESE CVCI**NGS**CFT IMTDGPSDGQ ASYKIFKIEK GKVTKSIELD APNSHYEECS<br>CYPDTGKVVC VCRDNWHGSN RPWVSFDQNL DYQIGYICSG VFGDNPRPND GTGSCGPVTS NGANGVKGFS<br>FRYGNGVWIG RTKSNSSRKG FEMIWDPNGW TETDSSFSVK QDIVAITDWS GYSGSFVQHP ELTGLDCMRP<br>CFWVELIRGL PKENTIWTSG SSISFCGVNS DTVGWSWPDG AELPFTIDK

Supplementary Table 2. % identity shared by CBC designs and WT N1 viruses[a]

Virus strain	rNA anti-sera					
	**NA5200**	**NA7900**	**NA9100**	**PR8/34**	**NC/99**	**Bel/09**
**PR8/34**	**87%**	**94%**	**92%**	**100%**	88%	83%
**USSR/77**	87%	**95%**	**92%**	**91%**	92%	84%
**NC/99**	85%	**93%**	**90%**	88%	**100%**	84%
**Bris/07**	85%	**92%**	**90%**	88%	**97%**	83%
**Bel/09**	**92%**	85%	**88%**	83%	84%	**100%**
**NIBRG-14**	**91%**	**87%**	**90%**	86%	85%	**90%**
**Sw/Bel/98**	**89%**	88%	**90%**	**85%**	**85%**	**94%**
**Sing/15**	**91%**	87%	**89%**	-[b]	-	**97%**

a. Percent identity was determined using the BlastP suite-2 sequences software. Numbers in bold identify where an IC_{50} of >4.3 (i.e. log_2 of dilution; 1:20) in the NI assay is observed.

b. "-" Analysis was not done for these sequence pairings.
